# GluR3B Antibody Was a Biomarker for Drug-Resistant Epilepsy in Patients With Focal to Bilateral Tonic-Clonic Seizures

**DOI:** 10.3389/fimmu.2022.838389

**Published:** 2022-04-06

**Authors:** Qingwei Lai, Qingyun Li, Xinyu Li, Heng Wang, Wei Zhang, Xiaotao Song, Peng Hu, Ruiqin Yao, Hongbin Fan, Xingshun Xu

**Affiliations:** ^1^ Department of Neurology, the Second Affiliated Hospital of Soochow University, Suzhou City, China; ^2^ Department of Neurology, Affiliated Hospital of Xuzhou Medical University, Xuzhou City, China; ^3^ Xuzhou Key Laboratory of Neurobiology, Jiangsu Key Laboratory of New Drug Research and Clinical Pharmacy, Xuzhou Medical University, Xuzhou City, China; ^4^ Institute of Neuroscience, Soochow University, Suzhou City, China; ^5^ Jiangsu Key Laboratory of Neuropsychiatric Diseases, Soochow University, Suzhou, China

**Keywords:** epilepsy, GluR3B antibody, drug-resistant epilepsy, focal to bilateral tonic-clonic seizures, prognosis

## Abstract

Considering the role of GluR3B antibody-mediated excitotoxicity in the progression of epilepsy, the purpose of this study was to evaluate the clinical significance of GluR3B antibody level as a novel biomarker for the prognosis of unknown etiology drug-resistant epilepsy (DRE) in patients with focal to bilateral tonic-clonic seizures. The study included 193 patients with focal to bilateral tonic-clonic seizures in the modeling cohort. Serum and CSF samples from patients were collected, and GluR3B antibody levels were detected by an ELISA kit. Serum and CSF GluR3B antibody levels in patients with DRE were significantly increased compared with those in patients with drug-responsive epilepsy. Univariate logistic regression analysis underlined that patients with high GluR3B antibody levels had a significantly increased risk of developing DRE. A logistic regression model demonstrated that increased GluR3B antibody levels were an independent factor in predicting DRE. External verification showed that the model constructed for the prediction of DRE had good adaptability. Finally, decision curve analysis highlighted the superior clinical net benefit in DRE prognosis by GluR3B antibody level. In summary, elevated levels of GluR3B antibody are an early biomarker to predict the prognosis of DRE; in addition, targeting GluR3B antibody may be a promising treatment strategy for patients with DRE.

## Introduction

Epilepsy is one of the most common serious neurological diseases. Currently, there are approximately 70 million epilepsy patients worldwide (1). Focal to bilateral tonic-clonic seizures are the common seizure type in adults (2). Because epilepsy is a chronic disease, epilepsy patients require long-term treatment with antiseizure drugs. Despite trials of multiple antiseizure medications, the seizures of approximately 30% of patients with epilepsy are still not effectively controlled, and these patients develop drug-resistant epilepsy (DRE) (3). It is difficult to predict and identify DRE early due to the complexity of the pathogeny. Thus, recurrent seizures of DRE usually cause an increase in the risk of psychological problems, cognitive disorders, declining quality of life, and even mortality (4). Early alternative treatments for DRE, such as epilepsy surgery, neurostimulation devices, and ketogenic diet therapy, have been shown to be superior to continuous administration of drugs (5–7). Therefore, the discovery of new biomarkers for the early prediction of DRE in patients would be beneficial in the choice of the optimal clinical treatment, thereby improving the clinical treatment effect and enhancing patients’ quality of life (8, 9).

Accumulated evidence has supported the autoimmune basis of DRE (10–12). In 2017, the International League against Epilepsy (ILAE) listed immune factors as one of the six major causes of epilepsy (13). Neuronal-specific autoantibodies are an important factor affecting the prognosis of patients with epilepsy, and patients with autoimmune epilepsy are prone to develop DRE (14–16). Among the known autoantibodies, GluR3B antibody was the first-discovered anti-neuronal autoantibody, and it specifically binds to amino acids 372-395 of the GluR3 subunit of the AMPA ionotropic glutamate receptor (17, 18). To date, some studies have shown that excitotoxicity induced by GluR3B antibody through the overactivation of the glutamate AMPA receptor damages neuronal cells and leads to brain injury (19, 20). Numerous studies have shown that GluR3B antibodies are not specific for Rasmussen’s encephalitis but are also present in some subtypes of intractable epilepsy (21, 22). Recent studies have shown that epilepsy patients positive for GluR3B antibody account for approximately 24% of patients with different types of seizures (23). Notably, GluR3B antibody was significantly associated with frequent seizures compared to drug-controlled seizures (21). Moreover, the seizure threshold was lowered and facilitated due to neuronal damage caused by GluR3B antibody (24). In addition, GluR3B antibody was closely related to the severity of epilepsy and mental, cognitive, and behavioral abnormalities in patients with epilepsy (23).

Although previous studies support the significance of GluR3B antibody in the progression of epilepsy, the role of GluR3B antibody in the prognosis of focal to bilateral tonic-clonic epilepsy remains unclear thus far. In this study, we evaluated the clinical significance of the GluR3B antibody level as a novel prognostic biomarker for predicting DRE in patients with focal to bilateral tonic-clonic seizures.

## Methods

### Patient Cohorts

Epilepsy patients hospitalized in the Affiliated Hospital of Xuzhou Medical University from January 2014 to October 2019 underwent extensive clinical examination, including full medical record evaluation, laboratory examination, electroencephalography (EEG) and magnetic resonance imaging (MRI). According to the criteria proposed by the International League against Epilepsy (25), we first screened 205 patients with focal to bilateral tonic-clonic seizures of unknown etiology from January 2014 to December 2017 as a modeling cohort. Thereafter, we screened 49 patients with focal to bilateral tonic-clonic seizures of unknown etiology from January 2018 to October 2019 as a validation cohort. The enrolled were newly diagnosed or monotherapy epilepsy patients. We collected a total of 254 fresh-frozen serum samples and 20 cerebrospinal fluid (CSF) samples. Written informed consent to participate in the study was obtained from all patients or their legal guardians before sample collection. Major exclusion criteria were patients with epilepsy related to structural abnormalities (hippocampal sclerosis, focal cortical dysplasia, brain malformations, cerebrovascular arteriovenous malformations, cerebral infarction, cerebral hemorrhage, craniocerebral trauma, and brain tumors, etc.), metabolic encephalopathy, inherited disease, severe cognitive impairment, the acute phase of central nervous system infection, autoimmune encephalitis (NMDA, AMPA1, AMPA2, LGI1, CASPR2, lgLON5, GABAB, GAD65, GlyR1, mGluR1, and mGluR5), demyelinating diseases, systemic lupus erythematosus, paraneoplastic syndrome, and psychogenic nonepileptic seizures. The patients were followed up for ≥ 24 months. The Morisky Medication Adherence Questionnaire (MMAS-8) was applied to evaluate the compliance of patients (26). MMAS-8 scores of < 6, 6-7, and 8 indicated poor, moderate, and high adherence, respectively. Drug-responsive epilepsy (n=166) was defined as a seizure-free period of at least 12 months or three times the longest seizure interval, whereas DRE (n=76) was defined as seizures at any frequency in the last 12 months after the administration of ≥ 2 drugs at optimal doses (27). Clinical data and serum samples as well as cerebrospinal fluid samples were collected. This study was approved by the Ethics Committee of Affiliated Hospital of Xuzhou Medical University (XYFY2016-KL017-02 and informed consent was obtained from each subject).

### Detection of GluR3B Antibody

Serum GluR3B antibody from epilepsy patients was examined in a blinded fashion by ELISA as described previously (23). Immunoplates (Nunc, Roskilde, Denmark) were coated with 100 µl per well of amino acids 372-395 of human GluR3B peptide with the sequence of NEYERFVPFSDQQISNDSSSSENR (20 mg/l, synthesized by Shanghai Qiangyao Biotechnology Company, Shanghai, China). Other plates were coated with 100 µl per well of phosphate-buffered saline (PBS) with 1% bovine serum albumin (BSA). Plates were incubated overnight at 4°C and then washed with PBS containing 1% BSA. To block nonspecific binding, 100 µl PBS containing 1% BSA was added to each well of the enzyme plates and incubated for 2 h at 37°C. After the microtiter wells were washed twice, serum samples from epilepsy patients were added to microtiter wells (100 µl/well) and incubated overnight at 4°C. After washing, 100 µl of horseradish peroxidase (HRP)-conjugated goat anti-human IgG (1:1000, Jackson Immunoresearch, West Grove, PA, USA) was added to each well of the GluR3B-coated and BSA-coated plates, and the plates were incubated for 2h at 37°C. Then, 100 µl peroxidase substrate (Kierkegaard and Perry Laboratories, Gaithersburg, MD) was added to each microtiter well. Optical density (OD) at 405 nm was measured using a microplate reader (Bio-Rad, Hercules, CA, USA). Repeated OD measurements were recorded 3 times. GluR3B antibody levels in each serum or CSF were determined using the following equation: [average OD of duplicate wells in the GluR3B-coated plate] - [average OD of duplicate wells in the BSA-coated plate].

### Statistical Analysis

The statistical analysis was performed by SPSS software (version 18.0, SPSS Inc., Chicago, IL, USA) and R (version 3.6.0). The nonparametric Mann-Whitney U test was applied to compare the average GluR3B antibody serum level between DRE and drug-responsive epilepsy. The clinical value of the GluR3B antibody level in discriminating DRE from drug-responsive epilepsy was determined by logistic regression analysis. Specifically, we constructed the full model (including the serum GluR3B antibody level as well as age, sex, epilepsy duration, age at first seizure, frequency of seizures before treatment, and initial therapy response) in the training data (n = 193) and validated the performance of the fitted model using the test data (n = 49) by calculating the area under the curve (AUC) of the ROC curve. The GluR3B antibody level with the maximum AUC was selected as the cutoff value. Afterwards, we transformed the GluR3B antibody level from a continuous variable into a binary variable in terms of this clinical value. Logistic regression models were further applied to evaluate the independent importance of the binary GluR3B antibody level while adjusting for age, sex, epilepsy duration, age at first seizure, frequency of seizures before treatment, and initial therapy response. Finally, decision curve analysis (DCA) was utilized to evaluate the clinical net benefit in predicting DRE according to Vickers et al. (28). The significance level was set to be 0.05 throughout our study.

## Results

### Baseline Clinical Data for Two Cohorts of Patients With Focal to Bilateral Tonic-Clonic Seizures

In the current study, there were 193 patients in the modeling cohort and 49 patients in the validation cohort with focal to bilateral tonic-clonic seizures. In the modeling cohort, 12 patients were excluded from the statistical analysis due to poor adherence to the prescribed medication or loss to follow-up. The clinical features of the patient cohort are summarized in **Table 1**.

**Table 1:** Clinical data of cohort patients.

Variables	Cohort, No. (%)		*P* Value
	Training cohort (n=193)	Validation cohort (n=49)	
Age, mean ± SD, y	34.1 ± 16.0	29.5 ± 16.9	0.074
Male	106 (54.9)	23 (46.9)	0.32
Female	87 (45.1)	26 (53.1)	
Epilepsy duration			
< 5 y	105 (55.4)	31 (63.3)	0.26
≥ 5 y	88 (44.6)	18 (36.7)	
Frequency of seizures before treatment			
≥ 4/year	26 (13.5)	13 (26.5)	0.026
< 4/year	167 (86.5)	36 (73.5)	
Age at first seizure			
≤ 12 y	32 (16.6)	10 (20.4)	0.53
>12y	161 (83.4)	39 (79.6)	
Initial therapy response			
Poor	60 (31.1)	14 (28.6)	0.73
Good	133 (68.9)	35 (71.4)	
GluR3B antibody			
≥0.325	70 (36.3)	19 (38.8)	0.75
<0.325	123 (63.7)	30 (61.2)	
Age at first seizure, y	27.2 ± 16.5	23.2 ± 15.8	0.13
Epilepsy duration, y	6.9 ± 8.6	6.3 ± 10.9	0.66
Number of AEDs	1.5 ± 0.6	1.5 ± 0.7	0.74

Our final study modeling cohort (n=193) included 60 patients (31.1%) with drug-resistant seizures and 133 patients (68.9%) with drug-responsive seizures. In the modeling cohort, we found that 70 (36.3%) patients had elevated levels of GluR3B antibody. Among them, the proportion of GluR3B antibody positive for newly diagnosed and monotherapy epilepsy were 26.9% (11/41) and 38.8% (59/152), respectively. The mean age at first seizure was 22.6 and 29.2 years, whereas the mean epilepsy duration was 10.1 years in patients with DRE and 5.5 years in patients with drug-responsive epilepsy. High frequency of seizures before therapy was present in 18 drug-resistant patients (30.0%) compared to 8 drug-responsive patients (6.0%). Poor response to initial therapy was present in 42 drug-resistant patients (70.0%) compared to 18 drug-responsive patients (13.5%). In this regard, the average numbers of drugs administered to drug-resistant patients and drug-responsive patients were 2.2 and 1.2, respectively. Levetiracetam (41.2%), oxcarbazepine (30.8%), carbamazepine (25.4%), valproic acid (22.5%) and lamotrigine (21.6%) were the most frequently used.

Our final study validation cohort (n=49) included 16 patients (32.7%) with drug-resistant seizures and 33 patients (67.3%) with drug-responsive seizures. The proportion of patients with GluR3B antibody positive was 38.8% (19/49). There were 7 (38.9%) newly diagnosed patients and 12 (38.7%) monotherapy epilepsy with GluR3B antibody positive. The mean age at the first seizure was 22.7 and 23.5 years, whereas the mean epilepsy duration was 9.3 and 4.8 years in patients with DRE and drug-responsive epilepsy, respectively. High frequency of seizures before therapy was present in 8 drug-resistant patients (50.0%) compared to 5 patients with a favorable drug response (15.2%). There were 11 drug-resistant patients (68.8%) with poor response to initial therapy compared to 3 drug-responsive patients (9.1%). Regarding medication, the average numbers of drugs administered to drug-resistant patients and drug-responsive patients were 2.3 and 1.1, respectively. Levetiracetam (44.6%), oxcarbazepine (29.5%), carbamazepine (25.0%), valproic acid (23.6%) and lamotrigine (18.4%) were the most frequently used.

### Patients With Drug-Resistant Seizures Showed Higher Levels of GluR3B Antibody

**Figure 1:**
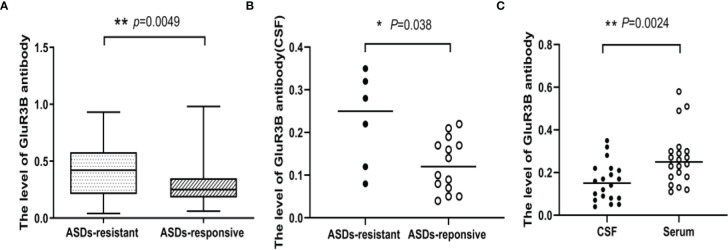
The level of GluR3B antibody was significantly increased in patients with DRE. Box plots representing the correlation of GluR3B antibody serum levels with patient response to antiseizure drugs **(A)**. Scatter plot representing the correlation of GluR3B antibody CSF levels with patient response to antiseizure drugs **(B)**. Scatter plot representing the level of GluR3B antibody in CSF and the serum of the same epilepsy patient **(C)**. *P < 0.05, **P < 0.01.

For the patients in the modeling cohort, serum GluR3B antibody levels were 0.40 ± 0.22 OD (95% confidence interval [CI] 0.35-0.46, n=60) in drug-resistant patients and 0.30 ± 0.18 OD (95% CI 0.27-0.33, n=133) in drug-responsive patients. Drug-resistant patients had much higher levels of GluR3B antibody than drug-responsive patients (*P=*0.0049, **Figure 1A**). Twenty patients had CSF samples. CSF GluR3B antibody levels were 0.23 ± 0.11 OD (95% CI 0.11-0.34, n=6) in drug-resistant patients and 0.12 ± 0.06 OD (95% CI 0.09-0.16, n=14) in drug-responsive patients. Similarly, CSF GluR3B antibody levels significantly increased in drug-resistant patients compared with drug-responsive patients (*P*<0.05, **Figure 1B**). In addition, we compared GluR3B antibody levels in CSF and serum samples from 20 epilepsy patients, and the results indicated that CSF GluR3B antibody levels (0.16 ± 0.10 OD) were lower than serum GluR3B antibody levels (0.27 ± 0.13 OD, *P=*0.0024, **Figure 1C**). In the modeling cohort, the proportion of GluR3B antibody-positive epilepsy patients with duration ≤2 years was 29.2% (21/72); the proportion of GluR3B antibody-positive epilepsy patients with duration >2 years was 40.5% (49/121). The level of GluR3B antibody in patients with duration ≤ 2 years and more than 2 years were 0.32 ± 0.21 vs 0.34 ± 0.20 (*P*=0.52). In order to understand the correlation between GluR3B antibody level and DRE in patients with the same duration of epilepsy and frequency of seizures prior to enrolment, we selected 56 patients with the same duration of epilepsy and frequency of seizures prior to enrolment, matched age and sex, including 28 patients with drug-resistant epilepsy and 28 patients with drug-response epilepsy. Serum GluR3B antibody levels were 0.42 ± 0.22 OD (95% CI 0.33-0.50, n=28) in drug-resistant patients and 0.21 ± 0.09 OD (95% CI 0.18-0.25, n=28) in drug-responsive patients. Drug-resistant patients had much higher levels of GluR3B antibody than drug-responsive patients (*P=*0.0003). Finally, the analysis of GluR3B antibody levels with epilepsy duration, ages at first seizure, frequency of seizures before treatment, as well as with initial therapy response didn’t highlight any significant association (data not shown).

### High GluR3B Antibody Levels Were an Independent Predictor of DRE

**Figure 2:**
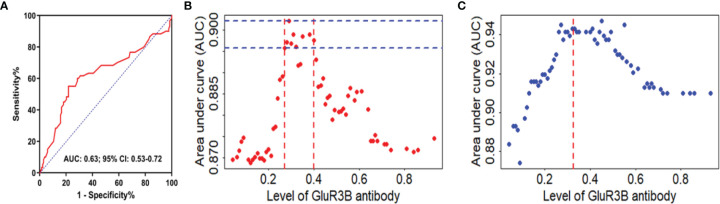
GluR3B antibody had good clinical value in discriminating DRE from drug-responsive epilepsy. ROC curve analysis of the GluR3B antibody level for the discrimination of DRE from drug-responsive epilepsy **(A)**. Different GluR3B antibody levels corresponded to areas under the curve (AUCs) of the full model in the modeling cohort **(B)** and validation cohort **(C)**. The mean GluR3B antibody level with the maximum AUC in the training data was 0.325. The GluR3B antibody level of 0.325 corresponded to AUCs of the full model in the validation cohort (red line, **C**).

**Figure 3:**
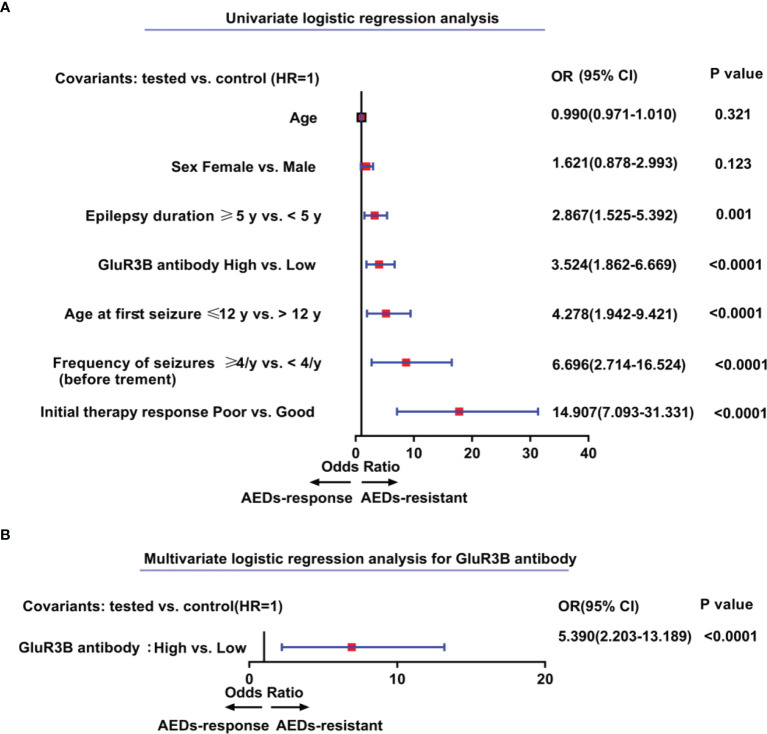
Elevated levels of GluR3B antibody represent independent predictors of patients’ higher risk for drug-resistant epilepsy. Forest plots of the univariate **(A)** and multivariate **(B)** logistic regression analysis for the prediction of drug-resistant epilepsy according to GluR3B antibody level. A multivariate model was adjusted for age, sex, epilepsy duration, age at first seizure, frequency of seizures before treatment, initial therapy response, and GluR3B antibody serum level **(B)**. 95% CI, 95% confidence interval; OR, odds ratio.

The clinical value of the GluR3B antibody level in predicting DRE was evaluated by ROC curve and logistic regression analyses. As shown in the ROC curve analysis (**Figure 2A**), the increased GluR3B antibody level showed moderate clinical value to discriminate DRE from drug-responsive epilepsy (area under the curve [AUC] 0.626, 95% CI 0.533-0.719; *P=*0.0051). Logistic regression analysis was used to evaluate the risk of patients with high GluR3B antibody levels developing DRE. First, we constructed the full model (including the serum GluR3B antibody level, age, sex, epilepsy duration, age at first seizure, frequency of seizures before treatment, and initial therapy response) in the training data and evaluated the performance of the full model by calculating the AUC of the ROC curve. Our data showed that the mean GluR3B antibody level with the maximum AUC in the training data was 0.325 (**Figure 2B**). Meanwhile, the GluR3B antibody level of 0.325 corresponded to the larger AUC of the full model in the validation cohort (**Figure 2C**). Therefore, 0.325 was used as an optimal cutoff value for the serum GluR3B antibody level. Afterwards, we transformed the GluR3B antibody level from a continuous variable into a binary variable (high antibody level and low antibody level) according to the cutoff value. As shown in **Figure 3A** by univariate analysis, patients with high GluR3B antibody levels (odds ratio [OR] 3.524, 95% CI 1.862-6.669; *P*<0.0001) had a significantly increased risk of developing DRE compared with patients with low GluR3B antibody levels. As expected, the univariate analysis confirmed the increased risk of developing DRE in patients with poor response to initial therapy (OR 14.907, 95% CI 7.093-31.331; *P*<0.0001), high frequency of seizures before therapy (OR 6.696, 95% CI 2.714-16.524; *P*<0.0001), ages at first seizure ≤12 year (OR 4.278, 95% CI 1.942-9.421; *P*<0.0001), and an epilepsy duration ≥5 years (OR 2.867, 95% CI 1.525-5.392; *P*=0.001) (**Figure 3A**).

To further evaluate the independent clinical significance of the GluR3B antibody level in predicting DRE, multivariate logistic regression analysis was performed (**Figure 3B**). Logistic regression models were adjusted for binary serum GluR3B antibody level along with patients’ initial therapy response, frequency of seizures before treatment, epilepsy duration, age at first seizure, sex and patient age. Indeed, logistic regression models highlighted the independent clinical significance of increased GluR3B antibody levels in predicting DRE (OR 5.390, 95% CI 2.203-13.189; *P*<0.0001) (**Figure 3B**).

**Figure 4:**
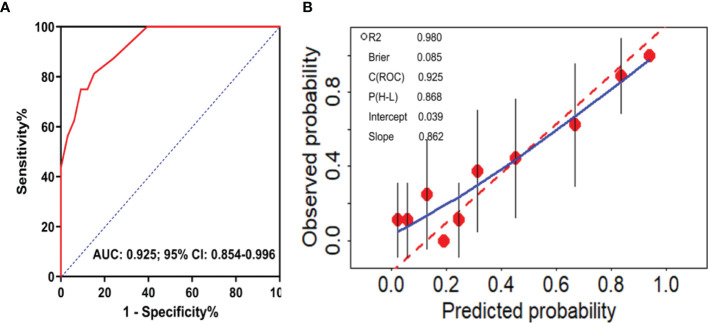
External validation of the predictive model of DRE. ROC curve analysis of the predictive model for the discrimination of DRE from drug-responsive epilepsy **(A)**. Calibration curve diagram to evaluate the prediction model of DRE **(B)**.

External validation was performed to evaluate the calibration and discrimination capabilities of this model by the Hosmer and Lemeshow test and ROC curve analysis. We successfully constructed a predictive model for DRE: y=1/(1+e^-z^). where y represents the probability of predicting the occurrence of DRE, e represents the exponential function and Z=-12.733+1.725*X_1_ +2.865*X_2_ +1.685*X_3_. X_1_, X_2_ and X_3_ represent frequency of seizures before treatment, initial therapy response, and the level of GluR3B antibody, respectively. The Hosmer and Lemeshow test was used to evaluate the calibration capability of the model in 49 patients from January 2018 to October 2019. The results showed that the *P* value was 0.868 (**Figure 4B**), indicating that the fitting effect was good. The AUC was used to evaluate the discriminative validity of the model, and the results showed that the AUC was 0.925 (95% CI 0.854-0.996, *P*<0.0001) (**Figures 4A, B**), suggesting that the predictive model of DRE constructed in this study had a good discriminative ability. Therefore, the model constructed for the prediction of DRE had strong external adaptability and could be a good tool to predict the development of DRE in clinical practice.

### Evaluation of GluR3B Antibody Levels Provided Clinical Benefit in Patients

**Figure 5:**
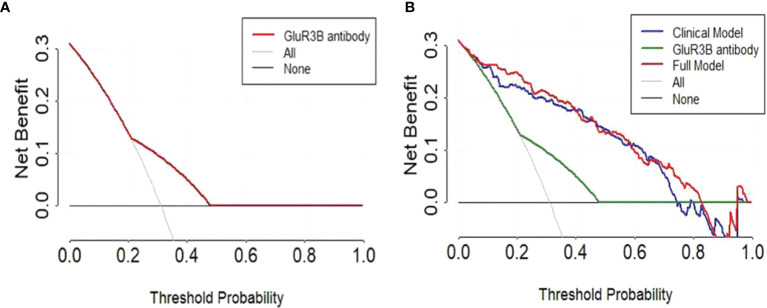
Decision curve analysis highlighted the clinical net benefit in the prediction of DRE by evaluating the GluR3B antibody level. Decision curves of the univariate prediction models for patients with DRE based on GluR3B antibody levels **(A)**. Decision curves of GluR3B antibody, the clinical model and the full model incorporating the GluR3B antibody level for predicting DRE **(B)**.

The clinical significance of increased GluR3B antibody levels in predicting DRE prompted us to further evaluate their clinical benefit through decision curve analysis. As expected, the univariate analysis showed that the evaluation of GluR3B antibody levels had superior clinical net benefit in epilepsy prognosis (**Figure 5A**). Moreover, the level of GluR3B antibody had superior clinical value in the risk stratification of patients with DRE (**Figure 5A**). Finally, the multivariate logistic regression model incorporating increased GluR3B antibody levels also demonstrated superior clinical net benefit compared to the clinical model in predicting DRE and patient treatment management for threshold probabilities of 70%-80% (**Figure 5B**).

## Discussion

Previous studies have shown that autoantibodies are an important factor affecting the prognosis of patients with epilepsy (29, 30). Autoantibodies that are involved in epileptogenesis can increase the excitability of neurons and facilitate discharge activity (31). Therefore, there is an increasing need to evaluate the role of autoantibodies in refractory epilepsy and their effect on disease treatment. This study evaluated the clinical value of the GluR3B antibody level as a novel biomarker for predicting DRE in patients with focal to bilateral tonic-clonic seizures.

Numerous studies have confirmed that GluR3B antibody is present in epilepsy patients with different seizure types (21, 32). Focal to bilateral tonic-clonic seizures are the common seizure type in adults. Therefore, we paid attention to the role of GluR3B antibody in the prognosis of focal to bilateral tonic-clonic seizures. Although GluR3B antibody levels were reported to be increased in the serum of epilepsy patients compared with healthy controls (21), we first showed that patients with DRE had higher levels of GluR3B antibody in both serum and CSF than drug-responsive epilepsy patients. Furthermore, we compared the level of GluR3B antibody in CSF samples and serum samples from 20 epilepsy patients, and the results showed that the GluR3B antibody level in CSF was lower than that in serum. This finding was in line with those of previous studies in which GluR3B-antibody-positive patients had more intractable epilepsy and mental, cognitive, and behavioral abnormalities than GluR3B-antibody-negative patients (23). Therefore, we speculate that autoimmune GluR3B antibody in the blood may enter the central nervous system through the damaged blood-brain barrier and induce neuronal damage and seizures. However, the mechanism of the participation of GluR3B antibody in epilepsy progression needs further study.

Increasing evidence has shown that early age at onset, a long duration of epilepsy, high frequency of seizures before treatment, and poor response to initial therapy response increase the risk for developing DRE (33–35). In the present study, our findings showed that a high GluR3B antibody level increased the risk of developing DRE (OR 3.524, 95% CI 1.862-6.669). We further evaluated whether elevated GluR3B antibody levels were an independent risk factor for predicting DRE by using the multivariate logistic regression model and confirmed that increased GluR3B antibody levels had independent clinical significance in predicting DRE (OR 5.390, 95% CI 2.203-13.189). Finally, the decision curve analysis showed a net clinical benefit in epilepsy prognosis by evaluating the GluR3B antibody level, thereby supporting personalized precision treatment.

The notion that serum GluR3B antibodies were implicated in epileptogenesis is supported by several different lines of evidence: First, GluR3B antibodies were present in a substantial proportion of patients (approximately 25%) with different types of epilepsy (23); Second, GluR3B antibodies could activate AMPA receptors and at least partially mimic excess glutamate, resulting in excitotoxic cell death in the central nervous system by activation of the receptor ion channel or a complement-mediated mechanism (20, 36); Third, mice that produced high levels of specific GluR3B antibody (following immunization with the GluR3B peptide) had lower seizure threshold and developed motor impairments and abnormal behaviors (24). Moreover, elevated level of GluR3B antibody was found in patients whose epilepsy was their main disease, but not in patients whose seizure accompanied some autoimmune diseases (37). In addition, some epilepsy patients with positive GluR3B antibody were sensitive to nonspecific immunotherapy (38–40). However, some studies also proposed that that serum GluR3B antibody in patients with epilepsy was a non-specific epiphenomenon. For example, a case report showed that the use of intravenous gammaglobulines neither altered serum GluR3B levels nor had any effect on patient’s seizure frequency in a patient with refractory epilepsy and high serum GluR3B antibody (41). Moreover, GluR3 antibodies were only found in a small portion of patients with intractable epilepsy in a case study (42). In our study, high levels of serum GluR3B antibody are associated with drug-resistant seizures and increase the risk of developing DRE, thus supporting the epileptogenesis of GluR3B antibody. It is speculated that there are several reasons that may cause these differences between these studies. First, the enrolled patients have different types of epilepsy syndromes and seizures. Secondly, the reference standard for GluR3B antibody positive is not consistent.

Some studies have proven that GluR3B antibody acts as a glutamate receptor agonist, specifically binding to GluR3-containing AMPA receptors and killing neurons *via* the activation of receptor ion channels (20). Notably, our previous study found that GluR3B antibody induces cytoplasmic mitochondrial dysfunction by activating AMPAR and causes the apoptosis of oligodendrocyte precursor cells (OPCs) (43). Because there are synaptic and nonsynaptic connections between OPCs and neurons, OPC injury may greatly alter neuronal excitability and their networks (44). A previous study also demonstrated that GluR3B-antibody-positive mice were more susceptible to seizures, and seizure scores were significantly associated with GluR3B antibody levels (24). A recent study found that GluR3B antibody could induce reactive oxygen species and kill both human neural cells and T cells in patients (45). We speculate that neuronal, OPC and T cell damage induced by autoimmune GluR3B antibody may be the biological mechanism of GluR3B-antibody-induced DRE.

Based on our clinical findings, serum GluR3B antibody can be a prognostic or predictive biomarker of DRE and influence the decision making for those patients. For newly diagnosed epilepsy with elevated GluR3B antibody, one third of patients may develop to DRE patients. If those patients were poorly controlled by 2-3 monotherapies or combined treatments, neurostimulation devices, ketogenic diet therapy or epilepsy surgery should be considered because early alternative treatments for DRE, such as epilepsy surgery or neurostimulation devices, have been shown to be superior to continuous administration of drugs (5–7). In addition, immunotherapy can also be considered for newly diagnosed epilepsy patients with frequent seizures and high GluR3B antibody, due to well response of immunotherapy such as intravenous immunoglobulin (IVIg) or plasma exchange in epilepsy patients with GluR3B positive antibody (38–40). Our unpublished data in two cases with GluR3B antibody-positive refractory epilepsy showed that intravenous immunoglobulin significantly reduced the frequency of seizures in these patients. Therefore, the detection of GluR3B antibody in patients with refractory epilepsy could be beneficial to those patients and improve their quality of life

One of the limitations of our study is low CSF samples of epilepsy patients. As the numbers of patients with available CSF samples is low (n=20 in both DRE and drug responsive patients in the training cohort), this will limit the predictive power of the association between CSF GluR3B antibodies and DRE outcome. Moreover, the different treatment options could have contributed to the deregulation of GluR3B antibody level among the patients. In this regard, the inclusion of a control cohort receiving antiseizure medications for non-epileptic diseases would be clearly beneficial in future studies for the correlation of GluR3B antibody levels with treatment schemes, strengthening the DRE-driven nature of GluR3B antibody upregulation.

In summary, it is necessary to perfect the detection of GluR3B antibody for patients with drug-resistant epilepsy of unknown etiology. Our study demonstrated that the GluR3B antibody level is an independent prognostic biomarker for predicting DRE. GluR3B antibody could be a potential therapeutic target for DRE.

## Data Availability Statement

The raw data supporting the conclusions of this article will be made available by the authors, without undue reservation.

## Ethics Statement

The studies involving human participants were reviewed and approved by the Ethics Committee of Affiliated Hospital of Xuzhou Medical University. The patients/legal guardians provided their written informed consent to participate in this study.

## Author Contributions

HF and XX designed and supervised the study. QYL, XL, XS, and WZ performed the experiments. HW and PH analyzed and interpreted the data. QWL and XX wrote the manuscript. All authors contributed to the article and approved the submitted version.

## Funding

This work was supported by grants from Jiangsu Key Laboratory of New Drug Research and Clinical Pharmacy (KF-XY201904), Youth Medical Science and Technology Innovation Foundation of Xuzhou Municipal Health Commission (XWKYHT20210582), Innovation and Startup Training Program for College Students of Jiangsu Province (201810313098H), Shandong Provincial Natural Science Foundation (ZR2019ZD32), and the Priority Academic Program Development of Jiangsu Higher Education Institutions of China.

## Conflict of Interest

The authors declare that the research was conducted in the absence of any commercial or financial relationships that could be construed as a potential conflict of interest.

## Publisher’s Note

All claims expressed in this article are solely those of the authors and do not necessarily represent those of their affiliated organizations, or those of the publisher, the editors and the reviewers. Any product that may be evaluated in this article, or claim that may be made by its manufacturer, is not guaranteed or endorsed by the publisher.
